# A Comparative Psycholinguistic Study on the Subjective Feelings of Well-Being Outcomes of Foreign Language Learning in Older Adults From the Czech Republic and Poland

**DOI:** 10.3389/fpsyg.2021.606083

**Published:** 2021-02-17

**Authors:** Blanka Klimova, Marcel Pikhart, Anna Cierniak-Emerych, Szymon Dziuba, Krzysztof Firlej

**Affiliations:** ^1^Department of Applied Linguistics, Faculty of Informatics and Management, University of Hradec Kralove, Hradec Kralove, Czechia; ^2^Faculty of Business and Management, Wroclaw University of Economics and Business, Wrocław, Poland; ^3^Department of Organization Development, Cracow University of Economics, Kraków, Poland

**Keywords:** psycholinguistics, positive psychology, cognitive linguistics, applied linguistics, L2 acquisition, foreign language learning, elderly people, FLL

## Abstract

Positive psychology has recently seen unprecedented rise and has reached vast achievements in the area of quality of life (QoL) improvement. The purpose of this study is to show that there are different aspects of well-being that make healthy older people motivated to learn a foreign language at a later age. The research was conducted in the Czech Republic and Poland in two groups of learners aged 55 years and more. The experimental group consisted of 105 Czech respondents who were targeted with an online questionnaire with the aim to determine the level of FLL outcomes connected to QoL in healthy older adults in their L2 acquisition. The second experimental group (*n* = 100) was established of Polish seniors who attended similar language courses. The findings of the research clearly show that FLL has an irreplaceable role as one of several non-pharmacological strategies utilized to improve the aging process and reduce drawbacks of aging. The results indicate that seniors' overall satisfaction and subjective feelings of well-being are enormously high when attending foreign language classes at older age. FLL, therefore, creates an environment that can enhance QoL of older adults that can be supplemented by other means such as well-balanced diet, other social activities, sport and physical activity, music, or computer games. All these intervention methods can significantly improve QoL of older adults and the parties engaged and/or responsible for taking care of older generation should take it into serious consideration.

## Introduction

At present, there is not much research comparing foreign results in well-being outcomes of foreign language learning (FLL) among healthy older adults. The recent review paper of Klímová and Pikhart (2020), however, revealed that FLL might have a positive impact on the maintenance and/or enhancement of cognitive abilities irrespective of age. In particular, FLL courses seem to offer new opportunities to healthy seniors for socialization and integration into society, which may positively affect their overall well-being. Furthermore, the research showed that it was partly through the stimulation of social well-being that the cognitive effects of FLL might be observed. In summary, FLL can be potentially used as a useful and efficient tool to maintain or improve subjective feelings of happiness and thus improve the quality of life in the participants of these courses (Pikhart and Klimova, 2020). From the perspective of psycholinguistics, FLL stimulates particular brain areas that are connected to cognitive function and this stimulation has proved beneficial. Therefore, this present pilot study is a natural continuation of this review findings with an international comparison of the learning outcomes of older adults connected to their subjective feelings of motivation, well-being, positive feelings, i.e., areas that are connected to positive psychology.

A lot of research claims that—from psychological and sociological perspectives—foreign language learning plays an important role both for a personal and societal development (Verga and Kotz, 2013). Furthermore, it is also important—from an economic perspective—for country's economic growth on a global scale (Hardach, 2018). All these aspects are well-described and researched, however, the aspects that are more subjective—but also very important—such as a motivation, subjective feelings of well-being, or satisfaction, still need much deeper investigation. Most recent research has proved that FLL has become one of the tools for improving one's well-being in seniors, along with other tools that could also prove to be very helpful. Among other non-pharmacological tools that can be utilized for the improvement of QoL can be mentioned, apart from the traditional ones, sports activity (Cwirlej-Sozańska et al., 2018; Mirandola et al., 2020), diet (Barchitta et al., 2020; Granado-Casas et al., 2020), meditation (Jin et al., 2020), societal help and programmes (Gu et al., 2019), or quality of the environment, including in particular the elimination of environmental barriers (Cwirlej-Sozańska et al., 2020).

Well-being can be understood as a person's experience of health, happiness, and prosperity, which is reflected in good mental health, subjective high life satisfaction, a sense of meaning or purpose of life, and ability to reduce or manage stress efficiently (What Is Well-Being?, 2020). These aspects of well-being are also connected with the acquisition of a foreign language, which, apart from the cognitive gains, can contribute to person's social and emotional well-being (Phenninger and Polz, 2018; Pot et al., 2018).

Especially in the last decade, there has been ongoing effort to shift research in second language acquisition from focusing on learning outcomes to non-cognitive factors associated with FLL, such as engagement, sociability, perseverance, or self-reliance (Butler, 2019). Furthermore, it has been evidenced that their cognition and emotions are inseparable phenomena (Swain, 2013). Research (Klímová and Pikhart, 2020) also shows that both cognitive and non-cognitive functions in FLL significantly differ across generations. Although it is known that adults can master a foreign language even in later age, it was proved that cognitive functions decline with an advancing age (Murman, 2015). However, this does not discourage older people from learning a foreign language since FLL bring them other benefits, such as social inclusion (Narushima et al., 2018), reducing feelings of anxiety and depression (Dewaele and MacIntyre, 2014; Pot et al., 2017), finding new motivation (Pot et al., 2019), which is then reflected in their feelings of joy, happiness, and satisfaction (Dewaele et al., 2019). Klímová and Pikhart (2020) also claim that it is through the stimulation of social well-being that the cognitive effects of FLL might be observed.

On the contrary, younger adults, i.e., university students, are usually very good at achievement tests because their cognitive performance is relatively high, but their motivation to learn a foreign language is completely different when compared with older adults. Their objective achievement are much lower when compared with younger adults but, on the contrary, their motivation and their positive feelings reach much higher levels, again, compared with the younger adults. As the findings of a study by Kormos and Csizer (2008) reveal, the main motivational factor in older adults to learn a foreign language is related to their identification as successful language users. This has been confirmed by other studies in this field, such as Dewaele and MacIntyre (2014) or Belnap et al. (2016) from the perspective of positive psychology.

The purpose of this study is to show that different aspects of well-being make healthy older people motivated to learn a foreign language at later age. Therefore, well-being and subjective positive satisfaction in these older adults are the most important motivators for their FLL, and as a consequence, their QoL is significantly improved. FLL is, therefore, a prerequisite to enhanced QoL, and, conversely, QoL is the condition of FLL. In other words, FLL is a very efficient non-pharmacological tool to improve QoL, but QoL cannot be achieved just by FLL. This research is intended to be a pilot study as it brings preliminary findings that must be further verified on a large scale and in a wider, ideally global, context. It can still be considered as an important impetus for further research that is urgently needed also due to the fact that the European population is aging rapidly and any non-pharmacological intervention that can lead to slowing down of a cognitive decline and improving subjective feelings of well-being is needed.

## Materials and Methods

### Research Problem and Question

This pilot study aims to explore older people's motivation for L2 acquisition or FLL in a later age from the perspective of positive psychology, and verify that especially different aspects of well-being stimulate healthy older people to study a foreign language. The main research question is as follows: *How much is subjective feeling of happiness important for FLL in older adults?*

### Research Sample

The research sample consisted of two groups, specifically, the respondents from Poland (*n* = 100) who formed the second experimental, comparative group, and the group from the Czech Republic (*n* = 105) whose subjects formed an experimental group. The age of the respondents ranged between 55 and 80+ years. The respondents from the Czech Republic (CR) were mainly from the East Bohemia Region and the respondents from Poland (PL) came from all over the country. They all attend or have attended language courses privately or at the University of the Third Age (U3A) in the corresponding countries. Their level of foreign language ranged from the beginner level to upper-intermediate, according to the Common European Framework of Reference for Languages (CEFR, 2020), i.e., from A1 to B2+. The sample was created randomly by contacting the potential respondents over the Internet by an email indicating that they could take part in a survey if they were interested.

The idea of the research sample is based on the situation that there are seniors who still experience healthy aging, i.e., without any significant cognitive decline caused by dementia or Alzheimer disease, or any kind of serious mental issues, such as schizophrenia or depression. The selection of the candidates for the research was conditioned by this factor.

### Questionnaire Survey

The research was conducted as on on-line questionnaire that was distributed to the senior participants of various language courses in CR and Poland. The content of this questionnaire was adjusted according to a standardized questionnaire by Woll and Wei (2019). This questionnaire is a standardized questionnaire already tested in younger groups of learners. It has proved useful and reliable; therefore, it has been adopted it into this research and we looked for further implications that could be derived from the answers in connection with positive psychology. All the questions are carefully chosen to focus on the ideas we needed to obtain, including several filler questions to hide the underlying idea of the research. In summary, the research methodology this manuscript is based on already proved to be very reliable and generating significant findings in previous psycholinguistic research.

Apart from the four questions on the demographic data of the respondents, there were 20 main items focusing on senior's attitude to foreign language learning from the positive psychology point of view and the last item represented an open question enabling the participants to express their ideas, comments, opinions, or feeling about FLL or the language courses they attend or have attended. The authors designed the questionnaire so that it would not take up more than 10 min of the participants' attention. The data were collected from April 2020 till June 2020 on-line. The on-line questionnaire was created and distributed by Google Forms to collect the data from the respondents. The respondents were informed that the data were collected anonymously and only the time stamp was collected without any email or IP address of the respondent. The respondents were informed about the research orally or personally by the course tutors.

The questionnaire is a standardized, but a modified questionnaire created by Woll and Wei (2019) using a dozen of questions about the respondents FLL subjective feelings. The 6-point Likert scale is used by the questionnaire with these options:

- Strongly agree,
- Agree,
- Agree a little,
- Disagree a little,
- Disagree, and
- Strongly disagree.

The research was conducted in accordance with the Helsinki declaration, i.e., the respondents were informed about the purpose of the study, and about the possibility of withdrawing from participation in the research at any time, as well as giving informed and voluntary consent to participate in this research.

### Statistical Analysis

Descriptive statistics was used for the processing of data. The statistical results were reported in frequency tables. The parametric tests were used to evaluate the relationships between intensity of positive outcomes of learning and sex, age, level of education, number of languages learned, and the type of language, currently studied, for each country. Firstly, the mean of rating for each statement was calculated for two countries separately to find out if groups of Czech and Polish seniors differs. Further, within each sample, the *t*-Student test and ANOVA test with *post-hoc* (Dunn-Bonferroni) were used for testing the relation between categorical socio-demographic variables and ratings of statements. The null hypothesis assumes that the intensity in each group is equal—variables are independent. The alternative hypothesis is one-sided, assumes that the intensity in one group is higher or lower. The Spearman coefficient was used to assess the strength and direction of relationship between two ordinal variables. The level of statistical significance was 0.05, however, lower levels were reported (0.01, 0.001). The analyses were performed using the PQStat v.1.8.0.

### Research Limitations

The research was conducted in two relatively big groups (experimental *n* = 105, second experimental *n* = 100, totally *n* = 205), however, to obtain statistically more reliable data, it would be necessary to conduct similar research in a wider and, ideally, more international context, this research was limited to the Central European region only. Despite these limitations, the research yielded interesting and important results that should be further verified in a more international context, even on a global scale. Another limitation of the research is that the data presented in Table 1 are not fully complete because of the fact that three Czech respondents did not reveal their age, gender and place of residence, and 30 respondents did not reveal their level of education. However, this limitation is marginal and does not in any way distort the results as it is far beyond a statistical mistake. This limitation does not pose any discrepancy between the results obtained and the described situation in Table 1.

**Table 1:** Demographic characteristics of the Czech and Polish respondents.

	**Country**	
**Age**	**CR**	**PL**
	***n* (%)**	***n* (%)**
55–60 years	16 (15.5%)	62 (62.0%)
61–65 years	24 (24.3%)	28 (28.0%)
66–70 years	17 (16.5%)	7 (7.0%)
71–75 years	27 (26.2%)	2 (2.0%)
76–80 years	14 (13.6%)	1 (1.0%)
80+	4 (3.9%)	0 (0.0%)
**Sex**		
Women	66 (64.7%)	60 (60.0%)
Men	36 (35.3%)	40 (40.0%)
**Place of residence**		
Town/city	72 (70.6%)	84 (84.0%)
Village	30 (29.4%)	16 (16.0%)
**Level of education**		
Elementary education/Vocational school	1 (1.0%)	8 (8.0%)
Secondary education	14 (13.6%)	1 (1.0%)
High school or university education	60 (58.8%)	92 (92.0%)
**Number of languages learned**		
One	67 (66.4%)	70 (70.0%)
Two	18 (17.8%)	23 (23.0%)
Tree	16 (15.8%)	6 (6.0%)
Four	0 (0.0%)	1 (1.0%)
**The type of language, currently studied**		
English language	85 (63.4%)	77 (77.0%)
German language	26 (25.5%)	22 (22.0%)
Russian language	23 (22.5%)	24 (24.0%)
French language	7 (6.9%)	3 (3.0%)
Other language	7 (6.9%)	12 (12.0%)

## Results

Although the demographic data of the respondents do not have such a significant impact on senior's attitude toward FLL, they show certain differences between the Czech and Polish seniors involved in FLL. Firstly, 80% of the Polish respondents attending the foreign language course are between 55 and 65 years old, while the age range of the Czech respondents until the age of 75 years is more balanced (consult Table 1 below). This indicates that the Czech respondents are more active even in later age. The questionnaire does not reveal the reasons for this discrepancy.

Secondly, the demographic results reveal that in the foreign language courses females outnumber males in both countries. In CR, the language courses are attended by 65% of females and in Poland, the number reaches 60%. Thirdly, 92% of the respondents in Poland and 95% in CR attained either high school or university education. Finally, as expected, most of the respondents (71% in CR and 84% in Poland) came from town or city.

Table 2 provides the basic statistics for the Czech and Polish group. It also contains results of *t*-Student test comparing differences between the means in the samples. Interestingly, the Czech group express less agreement on positive outcomes than the Polish group, excluding statements considering health, traveling, life motivation, and enjoying learning a new language.

**Table 2:** The basic statistics for the Czech and Polish groups and differences in perceived outcomes.

**Statement**	**Country**	**Mean**	**Standard** ** deviation**	**Median**	**Differences significance** ** (*t*-Student test)**
1. Learning a new language improves my concentration	CR	4.65	0.99	4	*t* = −5.739 *p* = 0.000001^***^
	PL	5.38	0.76	6	
2. Learning a new language improves my memory	CZ	4.80	1.06	4	*t* = −4.172 *p* = 0.0002^***^
	PL	5.36	0.77	5.5	
3. Learning a new language improves my attention	CZ	4.69	0.96	4	*t* = −3.764 *p* = 0.0002^***^
	PL	5.20	0.90	5	
4. Learning a new language improves my health	CZ	4.13	0.94	4	*t* = −1.667 *p* = n.s.
	PL	4.38	1.12	4	
5. Learning a new language improves my creativity	CZ	4.42	0.99	4	*t* = −5.589 *p* = 0.000001^***^
	PL	5.16	0.84	5	
6. Learning a new language helps me find new friends	CZ	4.70	0.99	4	*t* = −4.132 *p* = 0.0001^***^
	PL	5.22	0.76	5	
7. Learning a new language helps me understand different cultures	CZ	4.93	1.05	4	*t* = −2.566 *p* = 0.01^*^
	PL	5.27	0.75	5	
8. Learning a new language helps me while traveling	CZ	5.26	1.05	6	*t* = −1.695 *p* = n.s.
	PL	5.49	0.80	6	
9. Learning a new language helps me with learning other things, too	CZ	4.49	0.92	4	*t* = −5.162 *p* = 0.000001^**^
	PL	5.13	0.80	5	
10. Learning a new language helps me while looking for life motivation	CZ	4.50	1.04	4	*t* = −1.138 *p* = n.s.
	PL	4.67	1.03	5	
11. Learning a new language helps me with finding the purpose of my life	CZ	3.97	1.09	4	*t* = −2.869 *p* = 0.004^**^
	PL	4.45	1.23	4	
12. Learning a new language is enjoyable	CZ	4.84	1.09	4	*t* = −1.379 *p* = n.s.
	PL	5.05	1.05	5	
13. Learning a new language brings me personal satisfaction	CZ	4.73	1.14	4	*t* = −4.142 *p* = 0.0001^***^
	PL	5.31	0.79	5	
14. Learning a foreign language brings me feelings of happiness	CZ	4.72	1.13	4	*t* = −2.452 *p* = 0.015^*^
	PL	5.10	1.03	5	
15. Learning a foreign language is stressful	CZ	2.43	1.23	2	*t* = −3.641 *p* = 0.0003^***^
	PL	3.13	1.40	3	
16. Learning a new language does not bring any benefits to me	CZ	1.91	0.91	2	*t* = 0. 564 *p* = n.s.
	PL	1.83	1.11	1	
17. Learning a new language can have a negative impact on me	CZ	1.64	0.72	2	*t* = −1.340 *p* = n.s.
	PL	1.82	1.08	2	
18. Learning a new language occupies a lot of my time	CZ	2.93	2.93	3	*t* = −1.547 *p* = n.s.
	PL	3.21	1.27	3	
19. Learning a new language is a positive motivation for me	CZ	4.63	1.11	4	*t* = −3.725 *p* = 0.0002^***^
	PL	5.18	0.94	5	
20. Learning a new language will be useful for me in the future	CZ	4.63	1.13	4	*t* = −3,617 *p* = 0.0003^***^
	PL	5.18	0.98	5	

* *p < 0.05*,

** p < 0.01, and

*** *p < 0.001*.

Table 3 below then provide the main results of the Czech experimental group and the Polish second experimental group, which are more or less similar. The results of both groups, if one considers only agree and strongly agree items at the level of 80%+, clearly indicate that FLL brings older people a lot of benefits, which contribute to their positive emotional and social well-being. This is especially reflected in their perception that FLL contributes to their personal satisfaction, feelings of happiness, and positive motivation. Moreover, the respondents felt that by learning a foreign language, they can concentrate more, and their memory is enhanced. The Czech group also appreciated the social aspect of FLL because they found new friends, while the Polish group thought that FLL enabled them to improve their creativity. Interestingly, both groups seem to be active travelers since they reported that FLL helped them while traveling and discovering new cultures.

**Table 3:** Results of the Czech experimental group and Polish second experimental group.

**Statement**		**Strongly agree**	**Agree**	**Agree a little**	**Disagree a little**	**Disagree**	**Strongly disagree**
1. Learning a new language improves my concentration	CR	32.4%	52.0%	14.7%	0.0%	1.0%	0.0%
	PL	50.0%	38.0%	11.0%	0.0%	1.0%	0.0%
2. Learning a new language improves my memory	CR	40.2%	49.0%	7.8%	2.0%	1.0%	0.0%
	PL	48.0%	43.0%	5.0%	4.0%	0.0%	0.0%
3. Learning a new language improves my attention	CR	34.3%	42.2%	23.5%	0.0%	0.0%	0.0%
	PL	44.0%	35.0%	17.0%	3.0%	1.0%	0.0%
4. Learning a new language improves my health	CR	13.7%	43.1%	31.4%	6.9%	4.9%	13.7%
	PL	15.0%	31.0%	40.0%	8.0%	3.0%	3.0%
5. Learning a new language improves my creativity	CR	24.0%	50.0%	22.0%	1.0%	3.0%	0.0%
	PL	38.0%	47.0%	11.0%	4.0%	1.0%	0.0%
6. Learning a new language helps me find new friends	CR	35.3%	46.1%	16.7%	2.0%	0.0%	0.0%
	PL	41.0%	29.0%	20.0%	0.0%	0.0%	0.0%
7. Learning a new language helps me understand different cultures	CR	46.1%	42.2%	10.8%	0.0%	1.0%	0.0%
	PL	40.0%	46.0%	12.0%	2.0%	0.0%	0.0%
8. Learning a new language helps me while traveling	CR	62.4%	29.7%	5.0%	2.0%	1.0%	0.0%
	PL	61.0%	32.0%	5.0%	1.0%	0.0%	1.0%
9. Learning a new language helps me with learning other things, too	CR	25.7%	51.5%	18.8%	4.0%	0.0%	0.0%
	PL	34.0%	45.0%	18.0%	3.0%	0.0%	0.0%
10. Learning a new language helps me while looking for life motivation	CR	27.5%	42.2%	23.5%	3.9%	2.9%	0.0%
	PL	27.0%	26.0%	37.0%	8.0%	2.0%	0.0%
11. Learning a new language helps me with finding the purpose of my life	CR	14.0%	40.0%	28.0%	7.0%	9.0%	2.0%
	PL	23.0%	27.0%	33.0%	9.0%	6.0%	2.0%
12. Learning a new language is enjoyable	CR	42.0%	46.1%	7.8%	2.0%	2.0%	0.0%
	PL	40.0%	35.0%	19.0%	4.0%	0.0%	2.0%
13. Learning a new language brings me personal satisfaction	CR	37.3%	44.1%	15.7%	1.0%	0.0%	2.0%
	PL	48.0%	40.0%	9.0%	3.0%	0.0%	0.0%
14. Learning a foreign language brings me feelings of happiness	CR	37.3%	42.2%	15.7%	2.9%	1.0%	1.0%
	PL	40.0%	40.0%	15.0%	2.0%	1.0%	2.0%
15. Learning a foreign language is stressful	CR	2.0%	2.0%	21.8%	9.9%	41.6%	22.8%
	PL	5.0%	8.0%	35.0%	20.0%	14.0%	18.0%
16. Learning a new language does not bring any benefits to me	CR	0.0%	2.0%	6.9%	9.9%	44.6%	36.6%
	PL	1.0%	3.0%	6.0%	11.0%	29.0%	50.0%
17. Learning a new language can have a negative impact on me	CR	0.0%	1.0%	2.0%	3.9%	47.1%	46.1%
	PL	2.0%	3.0%	2.0%	6.0%	42.0%	45.0%
18. Learning a new language occupies a lot of my time	CR	2.0%	16.7%	25.5%	14.7%	30.4%	10.8%
	PL	2.0%	12.0%	33.0%	23.0%	19.0%	11.0%
19. Learning a new language is a positive motivation for me	CR	36.3%	46.1%	12.7%	2.9%	1.0%	1.0%
	PL	38.0%	46.0%	12.0%	1.0%	2.0%	1.0%
20. Learning a new language will be useful for me in the future	CR	36.0%	37.0%	19.0%	5.0%	3.0%	0.0%
	PL	44.0%	37.0%	15.0%	1.0%	2.0%	1.0%

However, to obtain more meaningful data, the statistical analysis of the results was performed. The findings on the outcomes of language learning on mental well-being revealed a correlation of age, sex, education level, number of languages studied, and the currently studied language with the distribution of responses in the group of Czechs and Poles.

**Figure 1:**
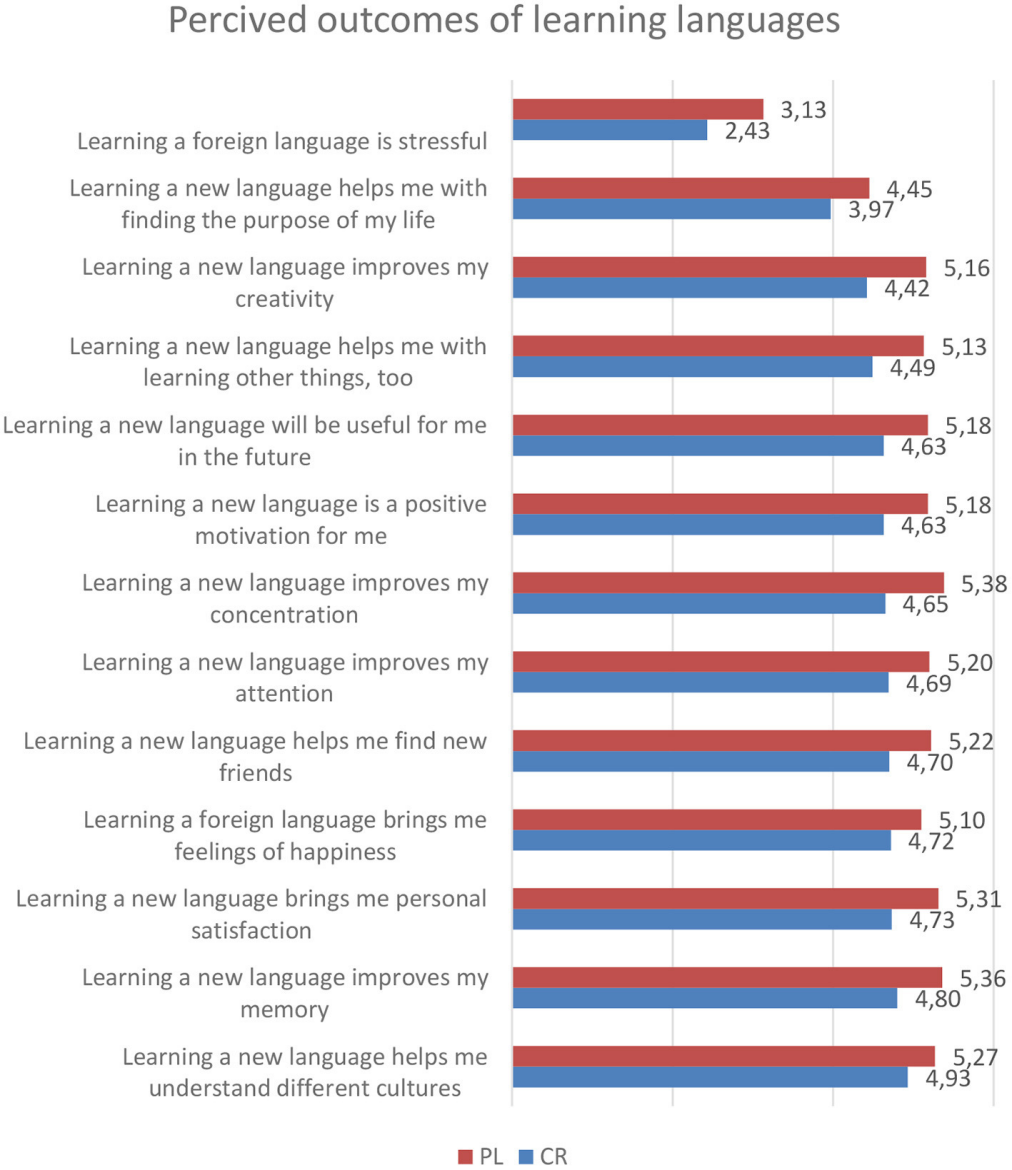
Perceived outcomes of learning languages in Czech and Polish group. The figure consists of the statements with significant differences in means between the groups.

The specific results of the analysis of the correlations between the perceived outcomes of language learning and age were in the group of Czechs as follows (Figure 1). There is a clear tendency indicating that perceived positive outcomes of decreased with age:

- Learning a new language helps me with learning other things, too *(r*_*S*_ = −0.21, *p* = *0*.039)
- Learning a new language is enjoyable (*r*_*S*_ = −0.24, *p* = 0.014)
- Furthermore, as age increased, the perception of negative outcomes increased:
- Learning a new language can have a negative impact on me (*r*_*S*_ = 0.20, *p* = 0.046)
- No statistically significant relationships were reported in the group of Poles *(p* > 0.05).

The analysis of the difference in perceived outcomes of language learning on well-being between **the sexes** revealed the following statistically significant relationships:

First, in the group of Czechs:

- Learning a new language helps me while traveling (*t* = 2.203, *df* = *90, p* = 0.033; Men M = 5,6, Women M = 5.1)
- Learning a new language brings me personal satisfaction (*t* = 2.338, *df* = *90, p* = 0.033; Men M = 5.1, Women M = 4.5)
- Learning a foreign language brings me feelings of happiness (*t* = 2.221, *df* = *90, p* = 0.028; Men M = 5,1, Women M = 4,5)
- Learning a foreign language is stressful (*t* = −2.221, *df* = *98, p* = 0.029; Men M = 2.1, Women M = 2.7)
- Learning a new language does not bring any benefits to me (*t* = −1.973, *df* = *90, p* = 0.051; Men M = 1.7, Women M = 2.1)^*^
- Learning a new language can have a negative impact on me (*t* = −2.537, *df* = *90, p* = 0.013; Men M = 1.4, Women M = 1.8)
- Learning a new language does not bring any benefits to me (*t* = 2.241, *df* = *90, p* = 0.016; Men M = 1.1, Women M = 4.4)

Second, in the group of Poles:

- Learning a new language improves my attention (*t* = −2.819, *df* = *98, p* = 0.001; Men M = 4.9, Women M = 5.4)
- Learning a new language helps me understand different cultures (*t* = −2.161, *df* = *98, p* = 0.033; Men M = 5.1, Women M = 5.4)

In both groups, the perception of positive outcomes of language learning was higher as the **level of education** increased.

First, in the group of Czechs:

- Learning a new language helps me while traveling *(r*_*S*_ = 0.27, *p* = 0.007).

Second, in the group of Poles:

- Learning a new language helps me while traveling, Poles *(r*_*S*_ = 0.21 *p* = 0.041)

The analysis of the difference in perceived outcomes of language learning on well-being between the **number of languages the respondents studied at the time of the survey** revealed the following statistically significant relationships. In the group of Czechs, the intensity of the positive outcomes decreased with the increase in the number of languages studied:

- Learning a new language improves my concentration *(r*_*S*_ = −0.22, *p* = 0.032)
- Learning a new language improves my attention *(r*_*S*_ = −0.21, *p* = 0.038)
- Learning a new language helps me with learning other things, too *(r*_*S*_ = −0.20, *p* = 0.049)
- Learning a new language is a positive motivation for me (*r*_*S*_ = −0.24, *p* = 0.018)

In the group of Poles, the following positive outcomes were statistically significant and positively related to the number of languages studied:

- Learning a new language improves my concentration *(r*_*S*_ = 0.24, *p* = 0.018)
- Learning a new language improves my attention *(r*_*S*_ = 0.22, *p* = 0.029)
- Learning a new language improves my creativity (*r*_*S*_ = 0.26, *p* = 0.008)
- Learning a new language helps me find new friends (*r*_*S*_ = 0.24, *p* = 0.017)
- Learning a new language helps me while traveling *(r*_*S*_ = 0.32, *p* = 0.001)
- Learning a new language helps me with finding the purpose of my life (*r*_*S*_ = 0.23, *p* = 0.024)
- Learning a new language is enjoyable (*r*_*S*_ = 0.39, *p* < 0.001)
- Learning a new language brings me personal satisfaction (*r*_*S*_ = 0.33, *p* = 0.001)
- Learning a foreign language brings me feelings of happiness (*r*_*S*_ = 0.36, *p* < 0.001)
- Learning a new language will be useful for me in the future (*r*_*S*_ = 0.22, *p* = 0.028)
- As the number of languages studied increased, the amount of stress experienced during learning decreased:
- Learning a foreign language is stressful (*r*_*S*_ = −0.23, *p* = 0.024).

A significant difference between the **language studied** and the experience of positive outcomes of learning was also reported in the group of Czechs.

First, in the group of Czechs:

- Learning a new language improves my concentration (*F* = 4.457, *df* = 53, *p* = 0.004, significant differences between English M = 5.0 compared to Russian M = 5.5)
- Learning a new language helps me with learning other things, too (*F* = 7.14, *df* = 53, *p* = 0.005, significant differences between English M = 4.70 compared to Russian M = 4.2)

In the group of Poles:

- Learning a new language improves my concentration (*F* = 2.738, *df* = 137, *p* = 0.031, significant differences between English M = 5.0 0 compared to Russian M = 5.5 and English M = 5.0 compared to German M = 5.5)
- Learning a new language improves my creativity (*F* = 2.528, *df* = 137, *p* = 0.048, significant differences between English M = 4.9 and Russian M = 5.5 and English M = 4.9 and German M = 5.5)
- Learning a new language helps me understand different cultures (*F* = 2.466, *df* = 137, *p* = 0.069, Eta-Square = 0.070, significant differences between English M = 5.1 compared to Russian M = 5.5 and German M = 5.0 compared to Russian M = 5.5)
- Learning a new language helps me with learning other things, too (*F* = 4.119, *df* = 53, *p* = 0.004 Eta-Square = 0.111, significant differences between English M = 4.9 compared to Russian M = 5.5 and German M = 4.6 compared to Russian M = 5.5).

The last question of the questionnaire was an open question so that the respondents could add any comment on learning a new language at older age, which they considered important. This question generated a vast number of answers as the respondents in both the experimental and the second experimental group were not limited by predefined questions of the questionnaire. There was no significant difference between the experimental and the second experimental group that could be analyzed in contrasting these two groups. The findings from this question revealed that the most important reasons to study a foreign language at such a late age were an internal feeling to start an activity that could potentially enhance cognitive functions, i.e., the seniors were aware of the fact that FLL could have a very positive impact on the improvement of their cognitive abilities. We believe this idea is not supported by any prior knowledge of some research into this issue, but it is rather intuitive and common-sense opinion. At least, no respondent mentioned that they had read about this enhancement of cognitive skills by FLL in literature. They rather highlighted the importance of learning new things that could improve their memory.

Another very important reason described by the respondents of both groups was improved social connections. Many of them, probably the majority in the experimental group, are attending these courses with their friends—peer pressure is still relevant here, i.e., many of them have friends who invited them to attend the courses even if they had been rather skeptical if it makes sense at such a late age. Social cohesion also plays an important part in their motivation to study at home as they sometimes meet for the purpose of studying at home. Naturally, this social activity enhances their well-being significantly as they do not spend their free time alone, but they can meet their friends instead, which on the other hand decreases their feeling of loneliness, depression, and anxiety.

Many of the respondents in both groups expressed their positive feelings connected to their FLL, i.e., their overall satisfaction with the course was not connected to their objective performance but rather to their subjective feelings of well-being and thus enhanced QoL. Some of them were aware of their cognitive limitations, as expressed in the last question, leading to their limited L2 acquisition. Many of the respondents expressed it, which means they are either well-informed or they subjectively experience this changed state of their brain and mind leading to significantly reduced ability of memory retention that is crucial in L2 acquisition. Their decline of cognitive functions is perceived subjectively as a reduced ability to acquire new vocabulary and therefore their performance in class is also influenced by this fact. This is the reasons why senior learners do not appreciate the fact when there are younger students in their classes as they can clearly see their progress and differences in L2 acquisition. On the other hand, the respondents expressed their satisfaction that the tutors are a generation, or two, younger than the respondents. The social encounter with the younger generation, if it is from the position of the student-teacher relationship, is stimulating. The respondents liked it because they can meet with new trends and ideas that are scarce in the context of other seniors.

## Discussion

The findings of this comparative pilot study indicate that FLL plays an important role in a normal aging process and could be considered a potentially useful intervention tool to improve well-being or even slow down the onset of serious cognitive impairments. This research proves that older people are stimulated to learn a foreign language by both cognitive and non-cognitive factors. Moreover, the results of this pilot study confirm Seligman's (2011) theory of well-being which claims that the aim of well-being is to enhance flourishing in everyday life by increasing positive emotions, engagements, meaningfulness, positive relationships, and accomplishment. These findings therefore support the idea that FLL may be an important contributor to the enhanced life experience and improve the positive feelings connected to an individual's subjective satisfaction. In addition, further research (James et al., 2011) also shows that more socially active older adults experience less cognitive decline in old age, which is a crucial aspect of FLL and naturally needs further investigation on a clinical level with trials. This is, however, a challenging task that will still need some time to accomplish.

In our research, despite the age of the respondents in both groups, many of them are very active and consider their FLL important for traveling and getting to know other cultures. In this study, these aspects were in both groups (experimental and second experimental) correlated with a higher education level. In fact, higher education plays a very important role in later life since another research (Fiske et al., 2009) indicates that the higher education level is one of the protective factors against depression. The findings of this study also revealed that FLL is more stressful and has a more negative impact on Czech women than men, while in the Polish group, it was just the opposite.

The results show that overall, the FLL courses are attended by more females than males. This gender difference is supported by the fact that women outlive men by 6–8 years (WHO, 2020). In addition, women tend to be more sociable and are more active in educational courses (Naud et al., 2019). Most of the respondents came from a city, where they have more transport and social opportunities to attend cultural, educational and other events, which increases also their chances for more quality living provided they feel secure in their city (Weziak-Białowolska, 2016).

Although numerous research shows that there may be both cognitive (Bubbico et al., 2019; Wong et al., 2019) and mental (Dewaele and MacIntyre, 2014; Pot et al., 2017, 2019) benefits for older people when learning a foreign language, the findings of this study reveal that age seems to be an important factor in the perception of FLL among older people. The results of the experimental group show that perceived positive learning outcomes, such as joy, decrease with age, while the negative benefits increase with age. There were no significant differences in the second experimental group. The reason may be that in the second experimental group of the Poles, the respondents' age ranged between 55 and 65 years mainly, while the Czech respondents were older, up to 75 years old. The findings of other research studies (Adams et al., 2011; Dotson, 2018) confirm that there are distinct differences in activity interests within specific age groups, which is also true for this study.

Furthermore, the results of the correlation analysis show that also the number of languages studied matters. While in the Polish group it was associated with many positive learning outcomes (concentration, attention, satisfaction, happiness, or finding the purpose of life), in the Czech group, surprisingly, it had several negative impacts (e.g., concentration or attention). Again, this might have been age-related perception since learning a totally new language becomes a very difficult endeavor when experienced in a later age. Both the experimental and second experimental group respondents confirm that FLL is both a time-consuming and painful activity. Despite this, they are still very motivated and from the positive psychology perspective, it is somehow surprising that an activity that is difficult and occupies so much of their free time can create such positive subjective feelings of satisfaction. One can only agree with Matsumoto (2019) who performed semi-structured interviews with third-age learners. Her findings show that FLL is clearly intrinsically rewarding for healthy seniors since it contributes to their sense of meaning in their life. As Viktorova (2020) puts it, it is a positive inner motivation that plays a significant role in seniors' FLL.

In addition, the teacher's role is also important in the FLL process since the teacher should consider the individual learner characteristics (Viktorova, 2020), his/her needs and cognitive abilities in order not to discourage older people from FLL and thus diminish their motivation. S/he should also know how to balance characteristic traits of each individual, e.g., assertiveness or passiveness. Research (Ramirez Gomez, 2016) confirms that seniors can learn a foreign language but must be taught in the ways that best reflect their cognitive strengths, such as the use of drilling exercises, scaffolding strategies, or providing written learning materials in a visible font.

The main limitations of this research study consist in its regional character. The research was conducted only in Central Europe. Therefore, to obtain statistically more reliable data, it would be necessary to conduct similar research in a wider and, ideally, more international context. On the contrary, the key strength of this study is objective processing of all collected data by using the correlation analysis whose results indicate that FLL contributes to overall well-being of older individuals and may improve QoL of aging population groups.

In conclusion, the findings of this pilot study showed that FLL may have an important role as one of non-pharmacological strategies in the aging process to keep the brain active. The results indicate that seniors' overall satisfaction and their subjective feelings of well-being are relatively high when attending a foreign language class at older age compared with more positive learning outcomes in younger older adults. FLL, therefore, creates an environment that may enhance QoL of older adults in a way that can be supplemented by other very efficient means such as well-balanced diet, other social activities, sport and physical activity, music, or computer games. All these intervention methods can be very efficient in improving QoL and the parties engaged and/or responsible for taking care of older generation should take it into consideration.

This paper claims that it is not very important to compare the level of improved well-being in the participants of other activities and classes for the elderly. It is more important that the research confirms that the well-being levels are dramatically improved in our participants regardless of the motivation behind them. We do not know what causes what and we know it is actually not important to look for the primary cause of the enhanced well-being. The mechanisms behind could be increased social activity, brain activity, etc., and all these contribute to the findings we have obtained. It is important to note the level well-being that have been obtained in the participants regardless of their study progress. It is enormous and goes beyond the traditional learning psychology findings that indicate that when the study results are good, then the motivation and satisfaction rise.

Generally, there is vast literature and research about cognitive decline in older people (James et al., 2011; Murman, 2015), however, more research and clinical trials are needed on the role of FLL in the maintenance or enhancement of cognitive functions so that we can obtain the data that could be utilized in the future fight with the new epidemy of dementia and Alzheimer's disease in the Western world. This pilot study should be an impetus for further research into the topic that is more than urgent and needed for the societal sustainability in the near future.

## Data Availability Statement

The original contributions presented in the study are included in the article/supplementary material, further inquiries can be directed to the corresponding author/s.

## Ethics Statement

All the respondents were the students of the U3A at the University of Hradec Kralove and Wroclaw University of Economics and Business. The participants' agreement with the intervention by the foreign language classes they attended was expressed by their involvement in those courses. All of the participants had signed the written consent with the language class intervention before the course itself. There was no experiment involving humans conducted, it was a regular language class. The data were collected by an anonymized questionnaire related to the participants' satisfaction with the courses, those that are conducted after all classes at the end of each semester. For this reason, there was no need to have an agreement of an ethics committee of the university. No personal data were collected and the questionnaire submitted to them does not demand any legal measures required by the GDPR regulation of the EU. All the participants provided their written consent before the language course had started.

## Author Contributions

BK, MP, AC-E, SD, and KF drafted, analyzed, wrote, and read the whole manuscript. All authors contributed to the article and approved the submitted version.

## Conflict of Interest

The authors declare that the research was conducted in the absence of any commercial or financial relationships that could be construed as a potential conflict of interest.
